# Effect of Fungicide Application on Lowbush Blueberries Soil Microbiome

**DOI:** 10.3390/microorganisms9071366

**Published:** 2021-06-23

**Authors:** Austin W. Lloyd, David Percival, Svetlana N. Yurgel

**Affiliations:** Department of Plant, Food, and Environmental Sciences, Dalhousie University, Truro, NS B2N 5E3, Canada; as979899@dal.ca (A.W.L.); David.Percival@Dal.Ca (D.P.)

**Keywords:** *Vaccinium angustifolium*, *Vaccinium myrtilloides*, fungicides, prothioconazole, chlorothalonil, soil microbiome

## Abstract

Lowbush blueberries (*Vaccinium* sp.) are perennial crops produced throughout eastern Canada and Maine through management of wild populations. Given the constraints of this cropping system, the application of fungicides is critical to reducing disease pressure and ensuring consistent yields. However, as plant health is intertwined with soil health, it is important to consider the impact of fungicides on microbial communities. To understand the effects of fungicides in this context, bacterial and fungal microbial communities from fungicide-treated plots, as well as untreated control plots (UTG) were analyzed using amplicon sequencing. The fungicides, considered collectively as a combined treatment group (CTG), lead to a loss in fungal richness. One family, Clavariaceae, had an increased abundance under prothioconazole relative to UTG. This finding may be significant as taxa in Clavariaceae have been thought to potentially form ericoid mycorrhizae with *Vaccinium*. Five functional pathways and 74 enzymes differed significantly in relative abundance between CTG and UTG including enzymes associated with soil nutrient cycles. Most notably, enzymes corresponding to the breakdown of halogen-organic compounds had an increased abundance in CTG, suggesting bacterial fungicide degradation. Some enzymes associated with soil nutrient cycles differed significantly, possibly implying changes to nutrient pathways due to fungicide treatment.

## 1. Introduction

Microbe-plant interactions play a critical role in the agroecological system, and understanding these relationships is a significant frontier in plant ecosystems management. These symbiotic relationships are the product of concurrent evolution between plants and microbes and are, in many cases, necessary to ensure that the plant will thrive [1]. Microbe–plant interactions may be particularly important in case of lowbush blueberries (a heterogeneous population consisting of *Vaccinium angustifolium* and *Vaccinium myrtilloides*) which are grown as a managed wild crop throughout the Canadian Atlantic Provinces, Quebec, and Maine. With over 67,000 hectares devoted to their production, lowbush blueberries are the most widely produced fruit crop in Canada by area of production [2].

Lowbush blueberries occur naturally on sandy acidic soils. Commercial production consists of managing pre-existing, wild populations and using practices including mechanical pruning and integrated pest management [3,4,5]. As lowbush blueberries are grown in their native habitat, spawned directly from wild populations, it stands to reason that managed lowbush blueberries may exhibit a particularly intimate relationship with the soil microbiome. Additionally, plants in the genus *Vaccinium*, as with many members of order Ericales, develop a distinctive mycorrhizal relationship with fungal species from the phyla Ascomycota and Basidiomycota known as ericoid mycorrhizae [6,7,8]. These fungi form coils of hyphae within the host root cell, with each cell being individually colonized from the root surface [8].

The crop is produced using a two-year cycle. After harvest, the field is mechanically pruned close to ground level and the crop spends the next year in a period of formation of new shoot uprights with floral induction and initiation occurring in midsummer [9]. The resulting floral bud growth and development occurring through to late autumn [9]. The following year, bloom occurs with floral densities in excess of 350 million flowers per hectare needing to be pollinated and fertilized [10]. The berries are typically harvested 70 days after anthesis, after which the management cycle repeats. Lowbush blueberry crops are managed with a number of fungicidal compounds in order to maintain a healthy canopy free of leaf disease pressures including Septoria leaf spot, blueberry rust, and Valdensinia leaf spot. Left unabated, these leaf diseases can cause extensive damage to the canopy resulting in premature defoliation, inadequate carbohydrate supply for plant growth and development, and significantly reduced berry yields [11]. While the effects of these chemical treatments on the plant itself have been well-studied, little is known about their effect on the plant microbiome. Given the interconnected nature of the blueberry and its native soil-combined with the potentially disruptive effects that these fungicides may have on the lowbush blueberry microbiome-developing a rigorous understanding of the effects that fungicides have on agriculturally-relevant microbes and the microbiome is crucial in advancing production techniques for this crop and in preserving this valuable natural resource.

The question of the effects of pesticides on the health of the soil microbiome is one of considerable environmental concern and has, as a result, been extensively studied. However, given the multiplicity of pesticides, both in terms of target organism (insecticide, fungicide, herbicide, etc.) and active ingredient mode of action, mobility, and persistence, it can be difficult to predict the effects of a given pest control product to a soil’s ecosystem. For instance, fungicides have been linked to an increase in soil organic matter and, as a result, microbial activity [12]. Conversely, the effect of fungicides on the health of the soil microbiome tends to be deleterious. The application of many fungicides resulted in decreased levels of soil biological carbon and nitrogen, and had variable effects on the nitrogen cycle depending on the environmental context of the experiment [13].

In order to further understanding of the ecotoxicology of fungicides in soil ecosystems, in general, and in the lowbush blueberry crop system, in particular, this study uses molecular genomics techniques to analyse the microbiomes of soils treated with two widely used fungicides, prothioconazole (Proline 480 SC) and chlorothalonil (Bravo 500) that differ in their mode of action and half-life in soil and are typically used to control leaf diseases. While work has been done on the subject of pesticide–microbiome interactions [13], given the context-dependent effects of pesticides on the soil ecosystem, it is important to investigate the way that specific fungicides affect specific agroecosystems. Given the potentially disruptive fungicidal effects, this study was aimed to evaluate the effect of prothioconazole and chlorothalonil, on the diversity, structure and function of soil microbiome. The choice of the fungicide was defined by their differences in mode of action, differences in uptake and mobility within plant tissue, and half-life in soil. Prothioconazole is an inhibitor of ergosterols has been demonstrated to have a short half-life of under 5.82 days [14,15], while chlorothalonil causes cell death by disrupting enzymatic action and has been demonstrated to persist in the soil and to lead to reduced levels of soil respiration 60 days after application [16,17]. Additionally, chlorothalonil has been found to be more prone to run-off compared to prothioconazole [18]. It was thus hypothesized that the two fungicides would induce significant changes to the fungal and bacterial microbiomes relative to control, and that the two fungicides would elicit significantly different effects from one another. Additionally, it was expected that the fungicide treatments may lead to changes in the function of the bacterial microbiome.

## 2. Materials and Methods

Site and Sampling: The soil samples were collected from a fungicide trial conducted in a commercial blueberry production field, in the first year of its crop cycle, in Highland Village, Nova Scotia (45.40406887, −63.6679066) on 28 August 2019. Soil samples taken on 1 June 2021 revealed the soil at the sampling site to be highly acidic with a mean pH of 4.98. Additionally, the field was low in organic matter with a mean organic matter content of 3.95%. Visual observations of the soil suggested it to be of a sandy character, and outside of the topmost centimeter, the soil possessed a light orange color. This observation conforms to the soil test results regarding organic matter and suggests a low retention rate of humic compounds. Soil surveys conducted by the Canadian government described the soil of the region as an orthic humoferric podzol and noted the soil of the region to be strongly acidic [19]. Soils such as these are typical of lowbush blueberry production, with sand and high acidity being common features of lowbush blueberry fields and acidity in particular has been associated with optimum plant growth in this context [20,21] For additional information about the characteristics of the sample site soil, see Table S1. Samples were taken from two fungicide treatment groups (prothioconazole, and chlorothalonil), as well as a control treatment to which no fungicide was applied. For each of the three treatments, there were four replications with each replication consisting of a 4 m × 6 m plot with a 2 m buffer strip separating each plot from the next. The fungicides had been applied to each of their respective plots three times prior to sampling—on 4 July, 15 July, and 26 July 2019. Prothioconazole (Bayer AG, Monheim am Rhein, Germany) was applied at a rate of 151 g a.i.·ha^−1^, while chlorothalonil (Syngenta Crop Protection AG, Basel, Switzerland) was applied at a rate of 3600 g a.i.·ha^−1^. Both fungicides were applied using a Bellspray Inc. Model GS hand-held sprayer unit (Bellspray Inc., Opelousas, LA, United States). Three samples of topsoil were taken from each plot for a total of 12 samples per treatment group. In total, 36 soil samples were acquired and kept on ice until they were returned to the laboratory. Upon arrival at the laboratory, each soil sample was sifted through a 2 mm sieve and then stored at −80 °C until DNA extraction could be performed.

DNA Isolation and Sequencing: For each sample, 0.250 g (wet weight) of sifted soil was used for DNA extraction. Extraction was performed using the Omega Biotek E.Z.N.A. Soil DNA extraction kit (Omega Bio-tek, Inc., Norcross, GA, United States) according to the manufacturer’s specifications. Extracted DNA samples were subsequently stored at −20 °C. Prior to sequencing, the extracted DNA was qualified with a NanoDrop 1000 spectrophotometer (Thermo Scientific, Waltham, MA, United States) to determine the concentration of the genetic material. 5 µL of each extracted DNA sample was sent to the Dalhousie University CGEB-IMR for library preparation and sequencing with the Illumina MiSeq platform (Illumina Inc., San Diego, CA, United States) with paired-end 300 + 300 bp reads, in accordance with the PCR procedure, primers, and sequencing details outlined in the Microbiome Helper protocol [22]. The DNA was sequenced for fungi-specific ITS2 genes (ITS2: GTGAATCATCGAATCTTTGAA forward primer, TCCTCCGCTTATTGATATGC reverse primer) as well as prokaryotic V6-V8 16S rRNA (16S: ACGCGHNRAACCTTACC forward primer, ACGGGCRGTGWGTRCAA reverse primer) [23,24].

Sequence Processing: The sequences were trimmed of their primers using QIIME2′s Cutadept plug-in [22,25,26]. The overlapping paired-end forward and reverse reads were stitched together using the QIIME2 VSEARCH wrapper [27]. Low-quality sequences were filtered from the dataset using QIIME2′s *q*-score-joined function (QIIME2 version 2019.7). Using QIIME2′s Deblur plug-in, the sequences were organized into amplicon sequence variants (ASV-high resolution genomic groupings [22,27,28]. In order to account for potential MiSeq bleed-through between runs (estimated by Illumina to be less than 0.1%), ASV which accounted for less than 0.1% of the total sequences were removed [22]. Taxonomic classifications were assigned to the ASV using QIIME2′s naïve-Bayes scikit-learn function, referencing SILVA databases (16S V6-V8, and fungi-specific ITS2) [28,29]. ASV that had a high probability of being the product of chimeric reads were removed from the dataset by filtering out any ASV which were unassigned at the division of level. Additionally, ASV assigned to mitochondria and chloroplasts were filtered out [22]. These ASV were used to construct two tables of ASV counts per sample—one each for the 16S and ITS2 datasets-along with a listing of all taxa present, and a phylogenetic tree for both datasets [22]. A preliminary analysis of 16S rRNA data revealed one sample from UTG to have much higher concentrations of both the phyla Firmicutes and Bacteroidetes compared to the other samples. As these phyla are heavily associated with fecal material [30], it was determined that this sample had likely been contaminated with fecal matter and it was subsequently discarded from both the 16S and ITS2 datasets.

Data Analysis and Statistics: QIIME’s diversity function was used to calculate both Shannon and Simpson’s indices (alpha diversity) as well as UniFrac matrices (beta diversity) for both datasets [31,32]. These UniFrac matrices were then subjected to an ADONIS test through which their values were fitted to a linear regression to determine what proportion of variance in community structure could be attributed to treatment. Principal coordinates analysis (PCoA) was performed using QIIME2 on the weighted UniFrac matrices. The UniFrac PCoA files were ported to RStudio (Version 1.2.5001, RStudio Inc., Boston, MA, United States) using the qiime2R package and plotted using ggplot2 [33,34]. Differential abundance analysis was performed with the STAMP software (version 2.1.3) using Welch’s t-test to identify taxa whose relative abundance varied significantly between treatments [35]. Adjusted *p*-values were calculated using the Benjamini-Hochberg FDR multiple-test correction.

Functional Potential Analysis: Using the 16S rRNA based ASV tables and reference sequences generated by QIIME2, functional potentials of the bacterial community were predicted using the PICRUSt2 software (version 2.3.0). Through this method, abundance tables were generated both for complete MetaCyc functional pathways as well as individual enzymes, categorized by Enzyme Commission (EC) numbers [36,37,38,39,40]. The relative abundances of these pathways and enzymes were tested for significantly differential abundance between treatment and group using the ALDEx2 package in RStudio [41,42,43]. This data was then graphically plotted using the ggplot2 package in RStudio [34].

In addition to being categorized by their specific treatment (prothioconazole, chlorothalonil, and untreated control (UTG)) and compared to each other (Treatment), the samples were further categorized into groups based on whether or not they had received a fungicide treatment. Both prothioconazole and chlorothalonil treatments were thus aggregated into a combined treatment group (CTG) to be compared to UTG group.

## 3. Results

### 3.1. Data Description

Analysis of 16S rRNA data revealed one sample from UTG to have much higher concentrations of both the phyla Firmicutes and Bacteroidetes compared to the other samples. As these phyla are heavily associated with fecal material, it was suspected that this sample had been contaminated with fecal matter and it was subsequently discarded from both the 16S and ITS2 datasets [44]. From the ITS2 dataset, two samples, both taken from the chlorothalonil treatment group, were deemed failed, as they contained fewer than 800 reads (416 reads and 140 reads, respectively). Those samples were discarded. From the 16S dataset, all noncontaminated samples were included. After the QIIME2 filtration processes had been performed, the two failed samples had been removed, the ITS2 dataset contained a total of 220,326 reads spread across 33 samples, with a mean per-sample frequency of 6677 reads/sample and a median frequency of 4137 reads/samples. For normalization purposes, the ITS samples were rarefied to a depth of 874 reads/sample, for a total of 28,842 reads. The 16S dataset consisted of a total of 835,577 reads across 35 samples, with a mean frequency of 23,874 reads/sample and a median frequency of 24,733 reads/samples. The 16S samples were normalized to a depth of 2400 reads/sample, for a total of 84,000 reads evaluated.

### 3.2. Overall Community Composition

**Figure 1:**
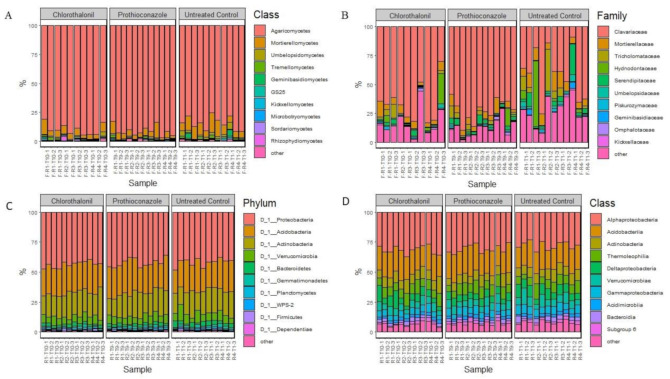
Microbial taxa identified in the study. (**A**)—fungal ITS2, class level; (**B**)—fungal ITS2, family level; (**C**)—bacterial 16S rRNA, phylum level; (**D**)—bacterial 16S rRNA, class level.

Basidiomycota was the major fungal division identified in our study and was represented by 88% of the total reads. Mortierellomycetes was the second most relatively abundant division represented by 7% of the total ITS2 reads, with the remaining 5% of reads being from the remaining divisions (Figure 1A. Within Basidiomycota, Clavariaceae was the most relatively abundant family, comprising 60% of the total reads, followed by Tricholomataceae, Hydnodontaceae and Serendipitaceae (4% of the total ITS2 reads each) (Figure 1B). Mortierellaceae was the most abundant Mortierellomycete found in the microbiome (7% of the total ITS2 reads).

The five most relatively abundant bacterial phyla—which comprised 95% of the total bacterial community—were Proteobacteria (43%), Acidobacteria (24%), Actinobacteria (19%), Verrucomicrobia (7%), and Bacteroidetes (3%) (Figure 1C). At the class level, the five most relatively abundant taxa were found to be Alphaproteobacteria (30%), Acidobacteria (20%), Actinobacteria (8%), Thermoleophilia (8%), Gammaproteobacteria (7%), in total comprising 74% of the total taxa present (Figure 1D).

### 3.3. Effect of Fungicides Application on Microbial Local Diversity

The Shannon diversity indices of fungal CTG was found to be significantly lower than those of UTG (*p* < 0.05) (Table 1). The Shannon diversity indices of the prothioconazole treated fungal community, when tested independently, was also significantly lower than those of UTG (*p* < 0.05), while the Shannon diversity indices of chlorothalonil treated fungal community was found not to differ from UTG (*p >* 0.05). Compared to UTG, CTG fungal microbiome had significantly lowered Simpson’s evenness (*p* < 0.05). However, neither fungicide, when compared independently to UTG, was found to be significantly different in its effect on fungal evenness (*p >* 0.05) and Simpson’s index variance between prothioconazole and chlorothalonil was not found to be significant (*p >* 0.05) (Table 1). Pairwise comparisons of Simpson’s evenness, and Shannon diversity indices did not reveal significant differences between CTG and UTG in bacterial alpha-diversity, as well as between prothioconazole and UTG, chlorothalonil and UTG, and between prothioconazole and chlorothalonil treated soils.

### 3.4. Effect of Fungicides Application on Communities Dispersion

Visualization of dissimilarity between fungal communities across treatments (prothioconazole vs. chlorothalonil vs. UTG) revealed limited clustering or visible trends in beta diversity (Figure S1A). The analysis of strength and statistical significance of sample groupings (ADONIS test) indicated that the treatments influenced the structure of the fungal community (R^2^ = 0.11, *p* < 0.05; Table 2). Additionally, comparing CTG to UTG indicated that 10% of fungal community variance can be attributed to whether a field was treated with fungicide (R^2^ = 0.099, *p* < 0.01). On the other hand, the type of fungicide (prothioconazole vs. chlorothalonil) was not a driving factor affecting fungal community structure (*p >* 0.1; Table 2). The fungicide treatments imparted a lesser effect on the bacterial community structure compared to the fungal microbiome. There was no visual separation between the treatment groups based on weighted UniFrac distances (Figure S1B), as well as no statistical significance of sample groupings into CTG and UTG, treatments, or prothioconazole and chlorothalonil was detected (Figure S1B; Table 2). However, the structure of bacterial communities treated with chlorothalonil differed significantly from UTG (*p >* 0.05), around 9% of community variation was explained by chlorothalonil treatment.

### 3.5. Effect of Fungicides Application on Soil Community Structure

The fungal family, Clavariaceae, was found to have an increased relative abundance in plots treated with prothioconazole compared to UTG (*p* < 0.05). Interestingly, this difference in abundance was not present in comparisons of chlorothalonil and UTG. The relative abundance of a member of Clavariaceae, *Clavaria sphagnicola*, was also increased in the prothioconazole treatment group (*p* < 0.05) compared to UTG (Figure S2). In comparing chlorothalonil and UTG, no taxa at any level of classification were found to differ significantly in their relative abundances (*p >* 0.05). Furthermore, no taxa were differentially represented between prothioconazole and chlorothalonil treatment groups (*p >* 0.05). In the 16S rRNA dataset, one bacterial genus, *Rudaea*, was found to be significantly more relatively abundant in UTG compared to CTG (*p* < 0.05). However, no taxa were found to differ significantly in relative abundance between the soils treated with either fungicide individually and UTG, nor did any taxa differ between prothioconazole and chlorothalonil treated soils.

### 3.6. Effect of Fungicides Application on Bacterial Functional Potentials

Based on 16S rRNA sequencing data, in total 2096 Enzyme Commission (EC) comprising 396 MetaCyc pathways were identified in the study. An analysis of the strength and statistical significance of sample function groupings (ADONIS test) indicated that individual treatments (prothioconazole vs. chlorothalonil vs. UTG) were not associated with differences in bacterial microbiome’s functional composition considering both individual enzymes (EC) and functional pathway (*p*-value > 0.05) (Table 3). However, when comparing pathway abundances by group (CTG vs. UTG), a small but statistically significant functional variation between CTG and UTG (R^2^ = 0.056, *p*-value < 0.05) was detected.

**Figure 2:**
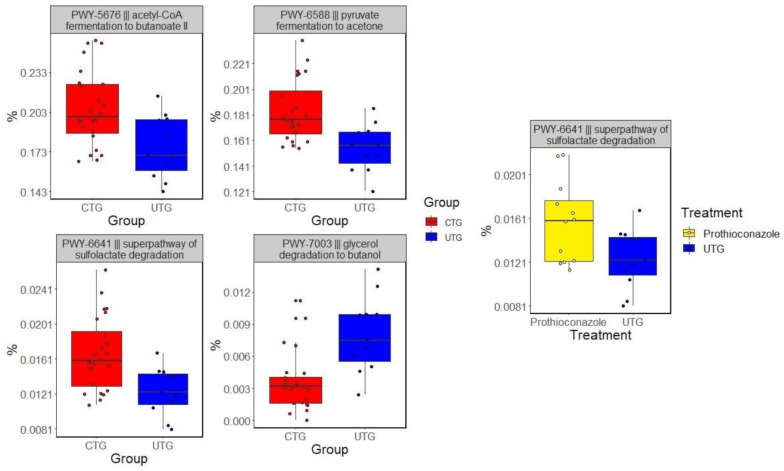
Pathways that were at differential relative abundances between CTG and UTG. Corrected *p*-values (*q*-values) were calculated based on Benjamini–Hochberg FDR multiple test correction. Features with (Welch’s *t*-test) *q*-value < 0.1 in ALDEx2 were considered significant and were thus retained. The analysis was based on 16S rRNA sequencing data.

Four predicted biological pathways were differentially represented between UTG to CTG (*p* < 0.1). PWY-5676 (acetyl-CoA fermentation to butanoate II), PWY-6588 (pyruvate fermentation to acetone), and PWY-6641 (superpathway of sulfolactate degradation) were significantly increased in their relative abundances in CTG relative to UTG. One, PWY-7003 (glycerol degradation to butanol), was overrepresented in UTG relative to CTG (Figure 2). PWY-6641 was also overrepresented in plots treated with chlorothalonil treated soils compared to UTG, while no pathways were found to differ significantly between prothioconazole treated soils and UTG.

**Figure 3:**
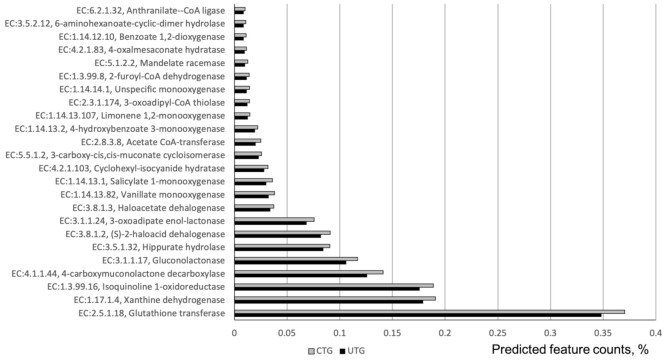
ECs involved in synthetic chemical degradation that were at differential relative abundances between CTG and UTG. Corrected *p*-values (*q*-values) were calculated based on Benjamini–Hochberg FDR multiple test correction. Features with (Welch’s *t*-test) *q*-value < 0.1 in ALDEx2 were considered significant and were thus retained. The analysis was based on 16S rRNA sequencing data.

In total 109 ECs were differentially represented between CTG and UTG. The 71 highly-abundant ECs are listed in (Table S2). Together they comprised around 3.5% of total predicted feature counts from 16S rRNA reads. A highly abundant enzyme, nitronate monooxygenase, EC:1.13.12.16 was significantly increased in CTG relative to UTG. Another potentially consequential enzyme to the processing of nitrogen in the soil is EC:1.17.1.4 (Xanthine dehydrogenase). Additionally, two enzymes, EC:3.8.1.2 and EC:3.8.1.3-(S)-2-haloacid dehalogenase and Haloacetate dehalogenase respectively were found to have significantly increased in abundance in CTG relative to UTG. 25 EC involved in xenobiotics biodegradation or metabolism were overrepresented in fungicide treated soil (Figure 3), including highly abundant CEs-Glutathione transferase (EC:2.5.1.18), Isoquinoline 1-oxidoreductase (EC:1.3.99.16), 4-carboxymuconolactone decarboxylase (EC:4.1.1.44), Gluconolactonase (EC:3.1.1.17), Hippurate hydrolase (EC:3.5.1.32), and 3-oxoadipate enol-lactonase (EC:3.1.1.24). They represented around 43% of predicted feature counts comprising all CEs differentially represented between CTC and UTG.

## 4. Discussion

Our data indicated that application of the fungicides prothioconazole and chlorothalonil had some effect on soil microbiome, but bacterial and fungal communities differed in their responses to the treatments, as reflected in the changes in the communities’ structure and/or diversity. When combined in one group (CTG), the fungal microbiomes from fungicide treated soils exhibited decrease in alpha-diversity and fungicide treatment was a driving factor affecting fungal community structure. However, no response of bacterial microbiome to fungicides treatments was detected. Interestingly, when compared to each other, despite their differing mechanisms of action and degrees of persistence in the soil, there was no significant difference between the two fungicides based on their overall effect on community structure (ADONIS test) and alpha diversity.

Considering individual fungicides, the treatment with chlorothalonil significantly affected both fungal and bacterial communities’ structure. Previous research on the effects of chlorothalonil application on bacterial communities have had mixed findings. In a 2019 study, it was found that the growth of 33% of the strains, isolated from the soils exposed to chlorothalonil, was affected by the presence the fungicide [45]. However, other trials have identified an increased growth in some bacterial taxa in the presence of chlorothalonil [46]. The effect of chlorothalonil on the soil microbiome was not surprising as chlorothalonil is persistent in the soil and has been shown to reduce soil respiration [17,46]. It was perhaps somewhat more surprising that prothioconazole, with its relatively shorter half-life, led to the loss in fungal richness as well as affected fungal community structure (ADONIS test). However, given that the fungicides are foliar treatments, in this context, it is perhaps not surprising that the two treatments did not yield more dramatic changes to soil microbial community structure relative to control as the amount of the fungicide-soil contact would not be expected to be large. An additional factor which may mitigate the effects of the two treatments on soil communities may be the physical and chemical characteristics of the soil itself. Given the coarse texture of the soil, its capacity for water retention is relatively low, and its low pH minimizes amount of adsorption in the soil matrix. It may be the case that these two factors combine to minimize the amount of time that any fungicide remains in the soil further reducing its impact on the soil microbiome.

*Clavariaceae* was found to be the most relatively abundant fungal family in all types of soils. The relative abundance of *Clavariaceae,* and more specifically *Clavaria sphagnicola,* was increased in prothioconazole treated soils. *C. sphagnicola* may be of agricultural relevance to the lowbush blueberry as it had been putatively associated with the formation of ericoid mycorrhiza associations (EMA) with plants in the genus *Vaccinium* and may exist in a symbiotic relationship with the blueberry plant [7]. One possible explanation for the expansion of *C. sphagnicola* may be that it fills niches which have been thinned out by fungicide treatment. If indeed *C. sphagnicola* is forming EMA with the blueberry crop, the increased abundance of *C. sphagnicola* may imply that it is replacing other symbionts or pathogens which are killed by the fungicide treatment, possibly implying a limited loss of overall mycorrhizal symbiosis. Alternatively, it may be the case that the plants, with the burden of disease lessened, have greater photosynthetic resources to spare and can support a larger number of symbionts per plant.

Analysis of functional compositions of soil microbiome identified four pathways differentially represented between UTG and CTG. Pathways PWY-5676, PWY-6588 and PWY-7003 were associated with anaerobic respiratory pathways. PWY-5676 and PWY-6588 were overrepresented in CTG, and PWY-7003 had an increased relative abundance in UTG. Superpathway of sulfolactate degradation, PWY-6641, was overrepresented in CTG compared to UTG. PWY-6641 is an umbrella category of three related pathways through which bacteria convert organosulfonate into sulfite and either pyruvate or acetyl-CoA. Additionally, PWY-6641 was found to have increased significantly in relative abundance in plots treated with chlorothalonil in comparison to UTG. The overrepresentation of this pathway may imply a change to the way in which sulfur compounds are processed in the soil induced by either chlorothalonil or fungicide treatment in general.

Variation in the relative abundances of a number of enzymes between UTG and CTG suggested changes in the function of bacterial microbiome under fungicide treatments. A highly abundant enzyme, nitronate monooxygenase, EC:1.13.12.16 was significantly increased in CTG relative to UTG. This enzyme is degrading aci-nitroethane into acetaldehyde converting a nitrogenous organic compound into an inorganic plant-available nitrogen compound [47]. Another potentially consequential enzyme to the processing of nitrogen in the soil is EC:1.17.1.4 (Xanthine dehydrogenase), which processes xanthine into urate [48]. The increase in the relative abundances of these enzyme under CTG may be an indicator of changes to the way in which the soil nutrients become available to the plants. Xanthine dehydrogenase is also involved in metabolism of pesticide ingredients. Additionally, two enzymes, EC:3.8.1.2 and EC:3.8.1.3-(S)-2-haloacid dehalogenase and Haloacetate dehalogenase respectively pertaining to the degradation of halo-organic compounds were found to have significantly increased in abundance in CTG relative to UTG [49,50]. This finding is significant in that both prothioconazole and chlorothalonil are themselves halocarbons, potentially alluding to the breakdown of pesticides by microbial action. Several other enzymes involved in synthetic chemical degradation and metabolism were found overrepresented in fungicides treated soils. These findings suggested a functional shift toward degradation of synthetic chemicals explained by the introduction of the fungicides into soil. Additionally, this apparent increase in bacterial degradation of fungicides may suggest the possibility of a reduction in fungicide effectiveness in certain contexts. If bacteria reduce the time in which the active ingredient is present, the total time during which the plant is protected from disease pressure is reduced. Indeed, it has been observed that the microbial selection process induced by repeated applications of a given fungicide may lead to an accelerated rate of biodegradation [51]. The increased abundance of these enzymes may be associated with this phenomenon and would therefore imply that the efficacy of the trial fungicides would decrease over time.

Despite some differences in relative abundances of several enzymes and functional pathways between CTG and UTG, where was a minor effect of fungicide treatments on the overall bacterial functional composition. While the analysis of the strength and statistical significance of sample function groupings did find that a small proportion of functional variation in pathways between CTG and UTG, comparing the individual treatment groups did not return significant results.

Considering EC abundances, no significant variation was found in response to either group or individual treatment. Some of the significantly differentially abundant enzymes mapped to reactions of potential ecological significance, suggest changes to the soil ecology as a result of fungicide application. Furthermore, though some variance in functional pathway abundances corresponded to fungicide intervention, the amount of variance was small enough that it is unclear whether fundamental changes to the soil’s ecosystem services would be expected as a result.

## 5. Conclusions

Given increased awareness of the importance of the soil microbiome on soil health and crop production, developing an understanding of the effects of agrichemicals on the soil microbiome is equally critical. While both fungicides evaluated in this study were shown to diminish soil fungal diversity, our findings suggest that the effects of prothioconazole may have a less deleterious effect on crop symbionts as shown by the increased abundance of a taxa of potential blueberry symbionts. Analysis of the bacterial microbiome did not indicate significant changes to the taxonomic profile but the predicted functions of the microbiome under treatment conditions relative to control did suggest the possibility of changes to soil nutrient processing and suggested the breakdown of fungicides by bacterial action.

## Figures and Tables

**Table 1:** Shannon richness and Simpson evenness of fungal and bacterial communities.

Category	Shannon	Simpson	Shannon	Simpson
	ITS2		16S rRNA	
UTG	3.769 ^A^	0.786 ^A^	9.217 ^A^	0.996 ^A^
Prothioconazole	3.158 ^B^	0.791 ^A,B^	9.133 ^A^	0.996 ^A^
Chlorothalonil	3.098 ^A,B^	0.789 ^A,B^	9.012 ^A^	0.995 ^A^
CTG	3.159 ^B^	0.790 ^B^	9.072 ^A^	0.996 ^A^

Significance of variance tested by Kruskal–Wallis test of alpha-diversity indexes by treatment and group. Within both ITS2 and 16S, variables with an ^A^ are significantly different from those with a ^B^; variables with ^A,B^ do not significantly differ from either (*p* < 0.05)

**Table 2:** Variation in amplicon sequencing sample groupings explained by weighted UniFrac dissimilarity.

Grouping	ITS2	16S rRNA
Treatment	0.113 *	0.073
Group (CTG vs. UTG)	0.099 **	0.051
Prothioconazole vs. Chlorothalonil	0.024	0.025
Prothioconazole vs. UTG	0.139 **	0.063
Chlorothalonil vs. UTG	0.101 *	0.092 *

Weighted UniFrac distances were calculated for each subset of samples. ADONIS tests were used to assess whether beta-diversity is related to sample groupings, 999 permutations, R^2^, ** *p* < 0.01 and * *p* < 0.05.

**Table 3:** Variation in amplicon sequencing sample groupings explained by weighted UniFrac dissimilarity.

Grouping	Pathway	EC
Treatment	0.078	0.088
Group (CTG vs. UTG)	0.056 *	0.068
Prothioconazole vs. Chlorothalonil	0.036	0.032
Prothioconazole vs. UTG	0.057	0.068
Chlorothalonil vs. UTG	0.083	0.101

PICRUSt2 pathway and CE tables were used with QIIME2 pipeline for ADONIS tests to assess whether their Bray–Curtis dissimilarity was related to sample grouping, 999 permutations, R^2^, * *p* < 0.05.

## Data Availability

The data presented in this study are openly available in NCBI sequence read archive under the accession numbers PRJNA731069.

## Supplementary Materials

The following are available online at https://www.mdpi.com/article/10.3390/microorganisms9071366/s1 Figure S1: Principal Coordinate Analysis (PCA) of microbial communities based on A-ITS2 (fungal) and B-16S rRNA (bacterial) communities Weighted UniFrac dissimilarity matrix, Figure S2: Eukaryotic taxa that were significantly overrepresented in comparison between prothioconazole and UTG, Table S1: Soil characteristics of sampling site, Table S2: ECs that were at differential relative abundances between CTG and UTG.
