# Supplementary material for: Effect of Fungicide Application on Lowbush Blueberries Soil Microbiome
Source: Microorganisms. 2021 Jun 23;9(7):1366. doi: 10.3390/microorganisms9071366 (PMC8305613; doi:10.3390/microorganisms9071366)
Supplement: Supplementary file 1 [file microorganisms-09-01366-s001.zip › Supplementary_Files/Fig_Sup_new.pptx]

## Slide 1
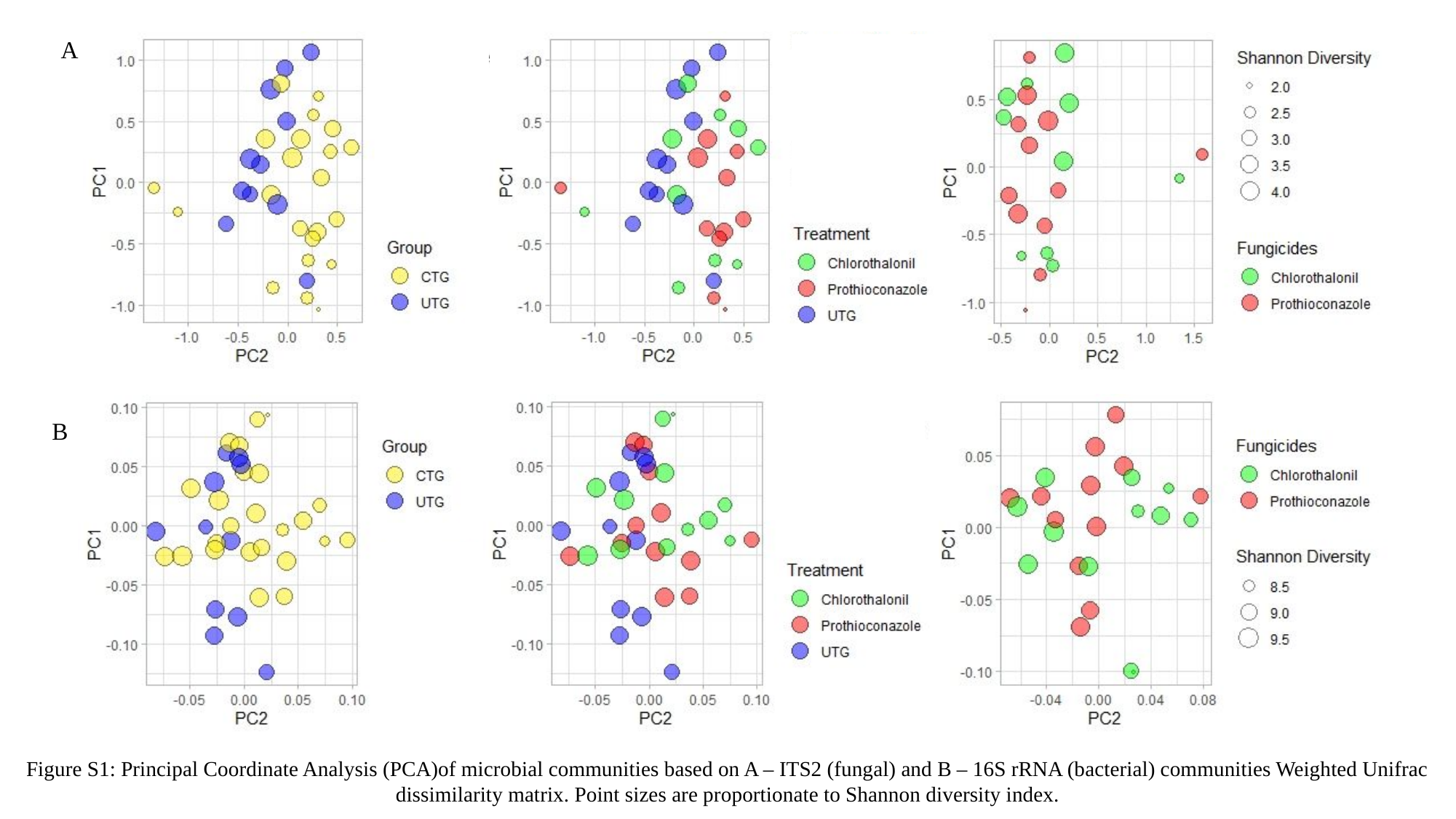

A
B
Figure S1: Principal Coordinate Analysis (PCA)of microbial communities based on A – ITS2 (fungal) and B – 16S rRNA (bacterial) communities Weighted Unifrac dissimilarity matrix. Point sizes are proportionate to Shannon diversity index.

## Slide 2
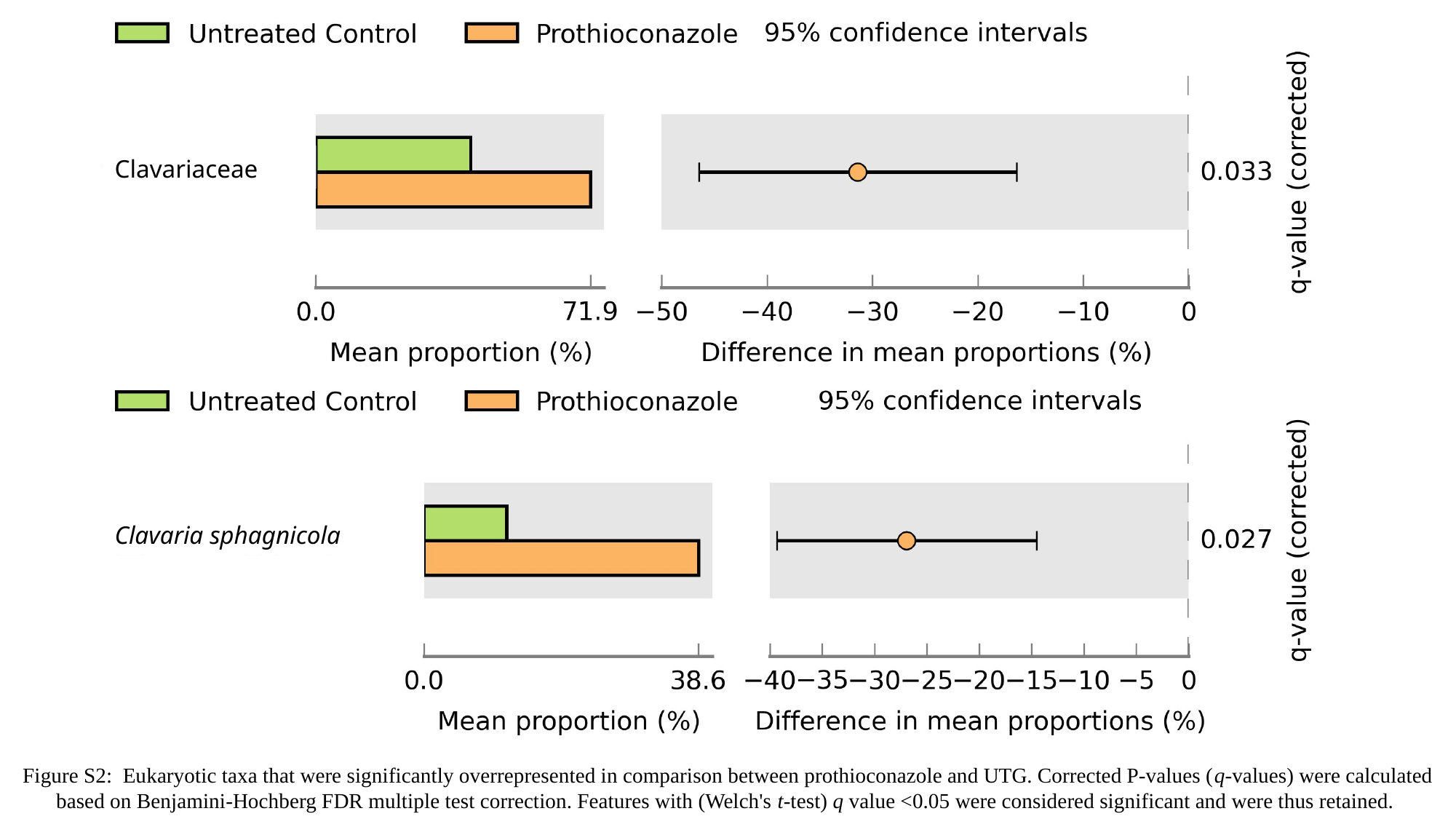

Clavariaceae
Clavaria sphagnicola
Figure S2: Eukaryotic taxa that were significantly overrepresented in comparison between prothioconazole and UTG. Corrected P-values (q-values) were calculated based on Benjamini-Hochberg FDR multiple test correction. Features with (Welch's t-test) q value <0.05 were considered significant and were thus retained.
