# Supplementary material for: Effect of Fungicide Application on Lowbush Blueberries Soil Microbiome
Source: Microorganisms. 2021 Jun 23;9(7):1366. doi: 10.3390/microorganisms9071366 (PMC8305613; doi:10.3390/microorganisms9071366)
Supplement: Supplementary file 1 [file microorganisms-09-01366-s001.zip › Supplementary_Files/Table_S1.pdf]

Table S1: Soil characteristics of sampling site

**Parameter**	**Value**
pH	4.98 ± 0.078
Organic Matter (%)	3.95 ± 0.071
P2O5 (kg/ha)	290 ± 67.9
K2O (kg/ha)	135 ± 24.7
Calcium (kg/ha)	170 ± 98.99
Magnesium (kg/ha)	35 ± 14.1
Sodium (kg/ha)	< 16
Sulfur (kg/ha)	78 ± 5.66
Aluminum (ppm)	1955 ± 33.9
Boron (ppm)	< 0.50
Copper (ppm)	0.23 ± 0.064
Iron (ppm)	116 ± 19.8
Manganese (ppm)	23 ± 8.5
Zinc (ppm)	1.22 ± 0.16
CEC (meq/100 g)	5.30 ± 0.283

Results taken from the average of two soil tests of bulked soil from sample site
